# Collectanea Medica

**Published:** 1814-10

**Authors:** 


### COLLECTANEA MEDICA,

CONSISTING OF

### ANECDOTES, FACTS, EXTRACTS, ILLUSTRATIONS, QUERIES, SUGGESTIONS, &c.

RELATING TO THE

*History or the Art of Medicine, and the Auxiliary Sciences.*

*Memoir upon the compound and smooth or simple Eyes of Insects, and on the Manner in which these two Species of Eyes concur in Vision.* By M. MARCEL DE SERRES, Professor of the Sciences in the Imperial University.*

Visus igitur quo insecta gaudent nulla penitus ratione cum nostris oculis aut cum camera obscura in qua rerum species, reflexionis ope, super charta aut panno albicante pinguuntur in comparationem venire potest.
SWAMMERDAM, *Biblia Naturæ*, tom. i, p. 502.

SIGHT seems to be one of the most perfect of the senses of insects; and in this respect these animals are in the first line among those without vertebræ, as birds are among those with vertebræ. Both enjoy a very long sight; and we know at how great a distance the maggot, like the bird of prey, perceives the object which it wishes to devour. Is the great quantity of air inhaled by these two orders of animals in the act of respiration, the cause of the acuteness of this sense, as it is of their muscular strength? or

* *Phil. Mag.* from *Magasin Encyclopédique*, Feb. 1814.

must we account for it from the great development of their retina and the largeness of their eyes? However difficult it may be to resolve a question abounding so much with difficulty, it is probable that insects and birds owe equally to the great abundance of air which they inhale, the acuteness of their senses, and the activity of their digestion; finally, the violence of their passions, if we may so express ourselves.

Far different from the vertebral animals in the structure of their eyes, insects enjoy the impression of external objects by means peculiar to themselves, and which furnish proofs that nature knows how to attain the same ends by very opposite ways. In fact, the operation of vision is not performed by insects in the same way as with most animals by the action of the luminous rays, which, after passing through the pupil, collect on the retina, but rather by the shaking of the optic nerves occasioned by the light which passes through the cornea. These eyes are constructed so as to receive the images of objects by the simple shock of the rays which these objects reflect, and this method of feeling must necessarily be very acute. Besides, insects not having, like the vertebral animals, pupils susceptible of contracting, it would seem that vision ought to be very perfect with them, on account of the great number of rays which fall continually on the facets of their eyes.

Nevertheless, one of the great inconveniences which results from the organization of insects, is the kind of immoveability of the parts in which their eyes are fixed. But nature has remedied whatever is unfavourable in this organization, by rendering the eyes of insects very much complicated, and by multiplying their facets in such a way, that one and the same eye presents as many as fourteen thousand, as Hooke observed in the *libellulæ*.* Nature has even sometimes multiplied the number of the eyes themselves, and this number seems to be always in the ratio of the size of these very eyes, and the immoveability of the parts in which they are situated. Thus, when the compound eyes are entirely wanting, the number of the flat or simple eyes is greatly increased, and certain species have thus even six pair of eyes. This is observed in some of the apteræ, many of the larvæ, and particularly in the spider. Indeed, the latter should be separated from the insects, as Messrs. Lamarck and Cuvier have already done; for their organization presents some very marked distinctions, and of the first importance. In fact, the spider, like the scorpion, has a kind of lungs, and consequently a real heart with blood-vessels; whereas, in the real insect, the organs of respiration are always ramified, and the vascular system, or at least what has been taken for it, never is. Sometimes, however, the eyes of insects are not situated in parts entirely immoveable, and certain species

* Swammerdam, *Biblia Naturæ*, tom. i. p. 490, and *Collection Académique*, p. 323. Hooke has given the eyes of the *Libellulæ* in his *Micrographia*, plates xxiii and xxiv.

present a kind of neck, which Comparetti* seems to have been the first to observe, and which permits the head to execute a variety of movements. This neck is very conspicuous in some of the carnivorous species, which not being able to pursue their prey on account of the disproportion of their organs of movement, require the faculty of seeing to a great distance. This organization is very evident in the *mantis*, the *empusa*, and the *mantispa*, in which the head may be directed to one side, or backward, and may even be twisted almost round. It may be remarked, in general, that the eyes are the more convex and the more salient, the more decidedly carnivorous the insect is, as the mobility of the parts in which they are situated, is always relative to the kind of life and to the habitudes of the insect. In short, we never saw the eyes of these animals present any kind of movement, and they always adhere to the parts where they are situated, and consequently are completely immoveable.

The sight is exercised by insects by two organs very different from those which perform this function in the vertebral animals and the *mollusci:* they exhibit even very striking differences from each other, as well in respect of their external conformation, as in their internal disposition. Some appear to be a large cluster of eyes united together, if we may so express ourselves, and others exhibit one only. The former have been called *compound eyes*, on account of their arrangement; and the latter smooth or simple eyes, on account of their simplicity. These two kinds of eyes concur to the same object, and are sometimes united on the same individual: thus the *orthopteræ*, like the *hymenopteræ*, present at once both compound and simple eyes, a disposition which is also observed in various kinds of *hemipteræ*, *neuropteræ*, and *dipteræ*. The other classes present only a single kind of eye; but, when there are only smooth eyes, their number is always considerable, probably in order to make up for their imperfection. In general, all winged insects have compound eyes, and this law does not seem to present any exception. In the *apteræ*, certain kinds present only compound ideas, such as the *cloportæ:* in truth, the latter seem formed by a collection of simple eyes. Other kinds have only simple eyes; such are the *julæ* and the *scolopendræ*, which present a certain number of simple eyes; but all these animals appear to me so different from the insect tribe in point of organization, that I have thought it right to pass them over.

With respect to the larvæ, those of half-metamorphosed insects uniformly have eyes similar to those of the perfect insects; whereas the larvæ of insects wholly metamorphosed have only simple eyes, which vary much in their number and position. The caterpillar has six or eight on the sides of the head. The false caterpillars, or the larvæ of common flies, have only two, like those of bees and the *stratyomæ*. Finally, a great many completely metamorphosed larvæ have no eyes at all.

---

* *Dinamica Animale*, part i. p. 103 to 106.

We

We may always remark, that the species which exhibit both compound and simple eyes (which has never been observed but in perfect insects) are those which have most need of seeing far, either because, by rising high in the air, it is requisite that they should distinguish their prey at a certain distance, or, because, having large flights to take, they should direct themselves with safety. In short, the insects of a great flight, like birds of prey, have very delicate and very extensive powers of vision

But in order to give an exact idea of the two kinds of eyes of insects, we shall describe each in particular, commencing with the compound eyes, the organization of which is most complicated, and terminating our description by the simple eyes. We shall afterwards endeavour to ascertain in what way the faculty of vision is executed among this order of animals, when we have followed up all the differences of organization which they present with respect to their eyes.

1. *Compound eyes.* The compound eyes are generally situated on the lateral or middle parts of the head, sometimes even completely at the base of it, and a few are placed near the antennæ, or more or less laterally and outside of these parts. Placed in orbitary cavities, they are protected not only by the sides of these cavities, sufficiently hard to oppose the impression of external bodies, and by their external membranes, which are almost as hard as the shelly envelope which covers the body, but also internally by soft parts, which seem to consist of tracheæ only. In the animals with vertebræ, the eyes are besides protected by several soft parts like the cartilages, the membranes of the eye-brows and eye-lids, which conceal them under a thick veil, and defend them from external objects. These parts, called by Haller *tentamina oculi*, are totally wanting in insects; and, besides, after knowing the conformation of their eyes, of what use could they be?

We must, however, observe, that some insects present in their cornea, and in general on the furrows which separate the hexagonal facets of this membrane, fine hairs, more or less long, and more or less thick. These have been regarded by Swammerdam and other anatomists as the eye-lashes of the eyes of insects, although they do not seem to perform their office: in fact, they appear to be so little essential, that they are wanting in the greater number; and when they are observed, they are always arranged on the lower part of the eye, a position which is far from being the most favourable to guard it against the admission of foreign bodies. Besides, scarcely any but the most glutinous bodies can adhere to the convex or polished surface of the eyes of insects; and, when they do, the animal can easily remove them with its fore legs. This is frequently observable in bees and flies, which take a pleasure in repeating the operation.

The situation of the eyes, or rather their position, is very variable in the different classes. As this position has a great influence in vision, we shall describe it in the chief families.

The

The most external membrane of the compound eyes is hard and transparent: it might therefore either be called the *sclerotica* on account of its hardness, or *transparent cornea* from its transparency. This last denomination agrees with it perfectly, for, as Swammerdam remarks, it has the flexibility, the firmness, and transparency of horn. This cornea or sclerotica, convex externally and concave internally, is formed by an infinity of hexagonal facets arranged with the greatest regularity alongside of each other. These facets, divided or separated by furrows which always follow the direction of the cornea, exhibit in some insects, the *hymenopteræ* in particular, hairs which resemble down. As to the lines or furrows of the facets of the cornea, they are curved and a little folded on account of the spherical convexity of the cornea, which interweaves in different places the hexagonal facets with the lines which separate them. In a word, the whole cornea is a true hexagonal net-work, the internal surface of which is divided into as many hexagonal facets as there are at the external surface.

Swammerdam thought that the cornea received some tracheæ, and that these constituted the hexagonal meshes of the cornea. He seemed to think also, that these tracheæ could in the eyes of the *nymphæ* serve for their expansion and unravelling. As to the first of these opinions, it does not seem to agree with the organization of eyes in general; for it is very rare that the tracheæ penetrate through the choroid and reach the cornea; and I only know one example, and that is the *libellula vulgaris*. The second fact, being only a consequence of the first, cannot be admitted, if it be true that the tracheæ rarely ever reach the cornea.

The form of the cornea necessarily determines that of the eye, and seems to have certain relations with the manner of living of the species. Sometimes this form varies in its proportions, and that in the species of one and the same genus; but it seems as if the *sphericity*, or the more or less angular form of this same cornea, is little subject to vary. In general, the cornea is the more spherical, and the more projecting, in proportion as the animal is carnivorous, or when the eye is concealed under a flap of the eye-lid, as we see in the *lampyræ* and others, where the sphericity of the eyes is so considerable, that it comprehends almost the whole head. We may also observe, that the smaller the compound eyes are, the greater is the convexity of the cornea. It is not the same with simple eyes, which in general vary little in point of size.

In order to give a precise idea of these variations, we have described them in a certain number of families, in order that their importance may be known. The cornea is in general cased in a cavity in the hard parts of the head. This union is so complete that it is frequently impossible to separate them: we might even doubt that any separation was possible, were it not for a small ring externally, which marks the line of adhesion. When the cornea is entirely separated from all the parts situated below its internal face, as if from its covering, it seems white and brilliant, which renders it similar similar to horn. This horn is very thick in certain species. It is transparent if we examine it externally; but, when it is not freed from the other parts situated below, it presents either bands or stripes of various colours, or a marbled appearance. It seems even completely black in a great number of species: most of the *coleopteræ, hymenopteræ,* and *lepidopteræ,* present this arrangement.

To conclude—this black colour and marbled tinge do not by any means belong to its texture, but depend, as may be easily ascertained, on the difference in thickness, and the various colours of the humour which adheres to it.

Under the cornea is seen a conduit or pipe, not very liquid, not soluble in water, and strongly adhering to this membrane. Its colour, in general, is between the darkest violet and the deepest black. When this coating presents this colour, which is most frequently the case, it is almost impossible to distinguish it from the humour of the choroid. This is not the case in the eyes, the coating of which is red or green, or of both colours united. We then see very distinctly the coating with its variegated colours, and the humour of the choroid with its black tinge, which never varies. This arrangement is very conspicuous in the *locusta gigantea, lilifolia,* the *libellula vulgaris,* and the greater number of the *tabani.* It is also very striking in the *gryllus lineola,* the eyes of which appear to be streaked with brown and green bands, and which is indebted for this singularity to the coating of the cornea being alternately brown and green, and that by nearly parallel bands.

It is, therefore, to the mixture of the tints of the tunic of the cornea, that the variegated colours presented to us by the eyes of insects are owing, precisely in the same way as, from its various degrees of thickness and colouring, the variegated stripes and marble tint arise, which appear at the exterior part of the eye, and which might have been thought peculiar to the cornea. Frequently, as a consequence of this arrangement, one and the same eye presents spots and stripes of various colours, or even one side of a colour totally different from the other.

The coating of the cornea, therefore, covers all the internal surface of this membrane. Its thickness and consistence, like its opacity, are very much subject to variation, as already observed; but it would seem that, in general, the more this tunic is opaque, the thicker and broader are the nervous threads which pass through it, in order, perhaps, that its opacity may not be an obstacle to vision.

We are under the necessity of anticipating a little upon our description, and of speaking here of the optic nerves, which, furnished by the retina, pass through the choroid and its humour, as does the coating of the cornea, in coming into correspondence with the facets of the latter membrane. This arrangement is not the same in the species which present bands or stripes, and in those which want them. If we carefully remove the cornea, and in such a manner as to remove very little of its coating, we observe, in the species in which the compound eye presents stripes externally, this coating to be composed of very distinct rays, one of which is blackish, and the other other much deeper, and so on alternately. These two colours are far from being uniform; and, however slightly we look at them, we soon perceive an infinite number of polygons, the centre of which appears to be white. All these white points are the extremities of the nervous filaments coming from the expansion of the grand optic nerve,* or from the retina, and which have traversed the choroid and its coating. We may convince ourselves of this in the following way: the brain being laid bare, we may follow the optic nerve, and see it directed to the eye, where it spreads, and is divided into a multitude of filaments. But if we draw these filaments, the white points disappear completely, and there only remain on the coating streaks variously coloured, which proves that these white points are the extremities of the nervous filaments. We may recognise this arrangement in the great species, such as the *libellulæ*, the *truxalæ*, and the common cricket. In the species which have no streaks on the cornea, we also observe on the coating of the cornea, the furrows or ridges which form the facets of this membrane, and the coating, which is more or less deep, having the form of a polygon, which represents the facets of the cornea.

These retinæ, peculiar to each facet, are those which Swammerdam has called the pyramidal of the eye. These compound fibres, according to him, proceed to a membrane as to a common centre, and it is the circular trachea from which the filaments issue: he also observes correctly, that it is through its substance that the tracheæ pass which ascend along the pyramidal fibres. The figure of these fibres is hexagonal, and their upper extremity is broader than their lower, spreading out a little, as it would seem, when they get into the concavity of the cornea, and taking the form of this membrane.

To conclude—Swammerdam states that he never was able to ascertain whether these fibres were muscular or nervous, although it would have been easy to convince himself of it, since they may be followed to the brain. As to the transverse fibres which Swammerdam has described,† I never perceived them. I am led to think that this expert anatomist must have made a transverse section in the eye and the brain, and that he formed these fibres by the section which he made in the optic nerve. At least, it is certain that these fibres communicate with the brain, for Swammerdam himself admits it: he remarks even that we might compare them to a very considerable nerve which we observe in the furrow, which nerve derives its origin from the brain.

In order to observe these white points, or the extremities of the nervous filaments which compose the particular retinæ of each facet, the cornea must be carefully removed, operating its section from

* We designate the nerve which proceeds to the compound eyes under the name of grand optic nerve, in order to distinguish it from the optic nerves which proceed to the simple or smooth eyes.

† *Biblia Naturæ*, tab. xx. fig. 5.

the the outside to the inside, and taking care not to remove the coating; for, however little we disturb the filaments, they are so contractile, that they close in upon the optic nerve, and no longer appear on the coating of the cornea. If, instead of dissecting the eye from the outside to the inside, we observe it by successively removing the internal parts, we can never discover this arrangement, even in the genera where it is most decided, such as the *gryllus,* the *mantis*, the *libellula,* and the *tabanus.* This depends on the contractibility of the optic nerves, which is sometimes so great as to draw the filaments even beyond the choroid.

Immediately under the optic filaments and the coating of the cornea we observe the coating of the choroid. This coating or varnish is a viscous substance, not liquid, soft, very clammy, and not very soluble in water. It is also strongly adherent to the membrane which it covers, when the latter exists. Swammerdam remarks, with great truth, that this opaque varnish stains the fingers like the common *pigmentum nigrum.* But this accurate observer seems to have confounded the coating of the cornea with the varnish of the choroid, when he says that the latter is variously coloured, according to the species. On the contrary, the black colour, and the opacity of the varnish of the choroid, are established beyond contradiction. This colour even seems to be internally connected with the texture of the choroid, for it is impossible to discharge it by repeated macerations. Contrary to the opinion of Hooke,* Swammerdam thinks that there does not exist in the eyes of insects any humour properly so called; and, in fact, neither the tunic of the cornea, nor the varnish of the choroid, deserves the name of humour, particularly if we compare them to what is understood by the term humour in the eyes of vertebral animals. Finally, the same anatomist regards the blackish varnish of the choroid as the extremity of certain fibres placed immediately under the cornea, fibres which have been torn on removing this membrane. This explanation appears very improbable, since, when the tunic of the cornea is of a colour different from the varnish, there never exist any blackish fibres corresponding with this same cornea. Besides, how could the colour of this varnish be always the same with that of the choroid? This single fact demonstrates, in my opinion, an analogy between the choroid and its varnish: it would even seem that the latter is produced by a kind of transudation which takes place through the meshes of this membrane itself.

If the varnish of the choroid be formed by a transudation of this

---

* Hooke and Boyle were the first who maintained that air was necessary to combustion and respiration, and that those operations consume but a certain portion of it. Hooke even conjectured that air was fixed in nitre, and that combustion was a chemical process, i.e. the solution of the burning body in a fluid, or its union with this substance. The chemists of the present day hold no other language. *Micrographia*, pp. 45, 104, 105.

membrane, it is evident that this varnish ought always to cover it, and this is precisely what is observed. But this varnish, as well as the choroid itself, does not always exist: it would seem even that all the species which shun the light are totally deprived of it. At least I have not observed it in the *blaps*, the *pedinus*, and most of the *tenebrios*: it is wanting beyond doubt in the *blattæ*, which, as is well known, are stupefied with the light of the sun. When the varnish and the choroid exist, it is not very difficult to separate this membrane from the varnish. It seems, then, to be most generally cellular; and in certain species, the longitudinal fibres of which it is composed are sufficiently decided to make it appear slightly streaked, on account of the tracheæ which are distributed over it: this is distinctly observable in the *truxalæ*. This arrangement has been well described by M. Cuvier, in his Memoir on the Nutrition of Insects, p. 42, tab. i. fig. 3. This organisation might induce a belief that the choroid is formed by the prolongation or union of the small tracheæ furnished by the large circular trachea: at least, all the tracheæ which proceed to this membrane are totally lost in it; and, as we cannot recover them, after they reach it, we ought to regard them as contributing to form the choroid. This membrane is therefore composed of a cellular texture tolerably close, on which there exists a collection of tracheæ furnished by the large circular trachea, and which are imbued with, or rather deeply penetrated by, a blackish varnish. The choroid is more or less black, but always opaque, and its sombre tint is as constant as its opacity. Finally, the longest macerations cannot make it lose its colour or opacity, as we have already observed.

(To be continued.)

